# Agile Application of Video Telemedicine During the COVID-19 Pandemic

**DOI:** 10.7759/cureus.11320

**Published:** 2020-11-04

**Authors:** Adeel Abbas Dhahri, Muhammad Rafaih Iqbal, Helen Pardoe

**Affiliations:** 1 Surgery, Princess Alexandra Hospital NHS Trust, Harlow, GBR

**Keywords:** telemedicine, virtual clinic, video telemedicine, video consultation, covid-19

## Abstract

Background

The coronavirus disease 2019 (COVID-19) pandemic led to a need to introduce video telemedicine for outpatients as an emergency measure without widespread stakeholder consultation. The patient and clinician experience of video outpatient consultation during the peak of the pandemic was studied for acceptability and to gather recommendations to improve the service during continuing infection control measures.

Methods

Outpatient video telemedicine was introduced over a 14-day period including the provision of equipment, systems integration and stakeholder communication. Patient and clinician experience were measured between 15 April 2020 and 5 May 2020.

Results

A total of 43 patients and 79 clinicians provided feedback. Of the patients, 86% were above the age of 30 years, with the largest patient group aged 51-70 years. Patient experience was positive. All (100%) patients found joining the video consultation easy; 93% of them recommended to use it for future consultations.

Clinician satisfaction was >90% with sound and video quality. Patients were less satisfied than clinicians in that they had communicated everything they wanted to (86% versus 95%). All (100%) patients thought that the video telemedicine solution met their needs, but 25% of clinicians believed that the patient experience of a video consultation was worse than a face-to-face clinic appointment. The three significant factors identified for introducing video consultations were successful IT, improved patient experience and digital healthcare records.

Conclusions

In the COVID-19 crisis, video telemedicine played a central role in outpatient consultations with excellent levels of success. With some differences in satisfaction level, clinicians significantly underestimate the level of patient satisfaction with outpatient video consultation.

## Introduction

The novel coronavirus disease 2019 (COVID-19) has changed the dynamics of delivery of healthcare across the globe when the World Health Organization (WHO) reminded healthcare authorities across the world to take specific measures to protect the community, patients and clinicians [1]. A study in China on the clinical characteristics of COVID -19 reported hospital-related COVID-19 transmission to be an essential contributing factor to a significant number of cases, suggesting rapid human-to-human transmission [2]. The need to reduce the contact and transmission of the disease led to the evolution of many ideas in which healthcare can be delivered remotely in a safe and effective way. This includes a change in the concept of outpatient clinic appointments to virtual clinics, thus reducing COVID-19 transmission risk. Recent studies on video telemedicine mentioned that solutions such as “Attend Anywhere” have wider acceptability to both clinicians and patients. It has been successfully evaluated not only in the United Kingdom (UK) but also around the world [3-5]. In the UK, National Health Services (NHS) has been using different forms of Technology Enabled Care Services (TECS) including telemedicine for more than a decade; however, widespread adoption in NHS district general hospitals has not been successful [6-10].

Telemedicine does not preclude the need for physical examination but helps in promoting healthcare services at a distance using technology at a lower cost. Different modalities under telemedicine range from text messages and emails, remote patient monitoring and teleconferencing [11,12]. Video telemedicine can effectively allow both clinician and patient with geographical separation to communicate using either smartphones or web-cam enabled computers. Additionally, it also enabled the monitoring of recently discharged patients.

Like every change, a video telemedicine solution has positive as well as negative effects. Positive influences include the establishment of trust between a patient and clinician, safety and convenience. The addition of the video element, to a virtual clinic, has the ability to pick up non-verbal communication, making the video consultation effective as face-to-face (F2F) consultations. Negative influences include patient suitability and preference, and the lack of physical examination or ability to deliver a procedure or intervention [13-15].

COVID-19 provided a unique opportunity to introduce a significant new way of working across the UK NHS. Through this study, we aim to understand the acceptability by both patients and clinicians and evaluate their experience to improve the service.

## Materials and methods

Setting

This qualitative prospective study was carried out for outpatient video consultations during the peak of the UK COVID-19 pandemic. In response to national guidance issued regarding outpatient consultations during COVID-19 [16], the hospital installed a video-enabled telemedicine solution in the outpatient clinics (oncology, diabetics, dermatology, respiratory, colorectal and general surgery) using IT customer relations officers to train clinicians in real-time (NHS Improvement supported system in our hospital Attend Anywhere). A quality improvement methodology was followed to successfully introduce the change utilizing Kotter’s eight steps for change to convert F2F outpatient clinic consultations to video conferencing telemedicine while assessing its suitability and acceptability by both the clinician and patient. Patients’ demographic details are not mentioned below to avoid a breach in Information Governance (IG). The effective outcome was assessed using the SWOT (strength, weakness, opportunities and threats) analysis methodology.

Kotter’s model for leading change

We used Dr. Kotter’s eight-step model of change methodology to lead and deliver the successful transition in our organisation [17].

Following the issuance of national guidance on stopping all non-essential F2F outpatient consultations, an utmost need of the hour was to create a mechanism for continued virtual outpatient consultations to deliver safe and effective care for both patients and clinicians. This requirement “created a sense of urgency” for the change to start (Kotter’s step 1).

A team of managers, clinicians and information and communication technology experts (ICT) was formed to “build the guiding coalition” (Kotter’s step 2). The team worked rapidly on the mechanism to deliver video conferencing. A high-level options appraisal was used to decide which video conferencing solution to implement for patient video consultations. A comparison made between different telemedicine options using score matrix and risks related to IG and associated with staff working from home analysed. After this brief comparison, “Attend Anywhere” was chosen as the video telemedicine solution because of broader applicability and quicker adaptation.

For a successful “development of a vision and strategy” (Kotter’s step 3), it was necessary to engage at many levels of the organisation to implement the change. Strategies were divided into leadership roles, implementation oversights, clinical processes, technical and testing requirements, staff training, booking processes, patient engagement and communication, IG perspective, IT equipment availability, and finance and procurement decisions. Under each of the work-streams in strategic planning, activities were identified and tasks divided between the team members.

At the start of the COVID-19 pandemic, a “volunteer army was ready to be enlisted” (Kotter’s step 4) as we took advantage of the national and international communication to inspire the wider team. Many routine hospital processes were suspended, leaving patients and staff available and motivated to help in response to the COVID-19 pandemic. We took advantage of the active situation, and a large, highly motivated group, including patients as volunteers, was engaged to focus on the need to deliver video outpatient consultations.

Kotter’s step 5 is to “enable action by removing barriers”. Once again, the opportunity of the COVID-19 pandemic emergency measures for management decisions, finance and IG processes led to streamlining of process within organisations, which successfully removed many obstacles previously encountered. Rapid approval from IG, clinical leads and patient representatives identified patient cohorts to prioritise. The video telemedicine was tested using “mock patients” in a clinical setting after confirming that technical requirements could be met from both hospital and patient location. Staff members included in design and testing were those managers, booking officers, nurses and clinicians involved in the outpatient setting as in pre-COVID era. Engaging the stakeholders in design and testing generated the early short-term wins for the project (Kotter’s step 6) to deliver successful consultations from locations within the Hospital Trust, consultants working from home as they were clinically shielding and patient group leaders.

Video outpatient consultation using telemedicine was successfully delivered and continues as part of the provision of routine services for patients to sustain acceleration (Kotter’s step 7). During the healthcare recovery phase of the COVID 19 emergency measures (three months after the institution of the emergency measures), consolidation and delivering more is the process of institutional change (Kotter’s step 8). The continued infection risks worldwide from COVID 19 supports the anchoring.

Duration

All consecutive video telemedicine consultations carried out over the three-week period (15 April 2020 to 5 May 2020) were included. The further qualitative survey of clinicians to gather information on influencing factors to continue to improve the delivery of video consultations to our patient population was administered from 10-17 July 2020.

Process

The patients were contacted to confirm if they were confident of receiving a video outpatient consultation. Patients who chose video telemedicine consultation were sent appointment letters along with a user guide. Information was added to the Hospital Trust website regarding video telemedicine, including links to joining a meeting, troubleshooting and user experience videos.

Inclusion criteria

The inclusion criteria included the following:

1. Patients above the age of 18 years 

2. Patients below the age of 18 years only if named guardian present during a consultation

3. Have a valid email address and access to a computer/smartphone/tablet with a camera and sound capability with Google Chrome or Microsoft Edge.

Exclusion criteria

The exclusion criteria excluded the following:

1. Patients under the age of 18 without a named guardian present.

2. Patients with no access to computer/smartphone/tablet.

3. Any appointment/consultation requiring “breaking bad news”.

After the video consultation, the patients were sent out a feedback form by email. The clinician doing the video consultation received an automatically generated feedback form. The second clinician information-gathering questionnaire was administered three months after the initial introduction of the video telemedicine.

The feedback questionnaire for both patients and clinicians are available in the supplementary data appendix.

## Results

Patient feedback

A total of 43 patients provided feedback. Most (n=37; 86.04%) of the patients were above the age of 30 years (Table 1).

**Table 1:** Patient age distribution

Age, years	Number (n)	Percentage (%)
Under 18	5	11.62
19–30	1	2.32
31–50	10	23.25
51–71	20	46.50
71+	7	16.27

Mobile (n=17; 39.53%) and laptop (n=14; 32.55%) were the two most commonly used devices. Google chrome and safari were the most commonly used browsers. Most of the patients were able to access the video consultation by themselves (n=40; 93.02%), whereas only a few required help (n=3; 6.97%). All the patients found joining the video call easy (very easy: 35/43; easy: 8/43). Majority (n=40; 93.02%) of the patients of the patients opted to choose video consultation for future again, whereas none of them opted not to choose it again (Table 2).

**Table 2:** Patient response to "How likely would you choose the video consultation again"

	Number (n)	Percentage (%)
Very likely	36	83.72
Likely	4	9.30
Neither likely nor unlikely	3	6.97
Unlikely	0	0
Very unlikely	0	0

Majority of the patients were pleased with the sound quality (33; 76.74%) and the video quality (34; 79.06%) (Table 3).

**Table 3:** Patient/clinician experience Data are presented as n (%).

		Very Good	Good	Unsure	Poor	Very Poor
Patient (n = 43 )	Sound quality	33 (76.74%)	10 (23.25%)	0 (0%)	0 (0%)	0 (0%)
	Video quality	34 (79.06%)	8 (18.60%)	1 (2.32%)	0 (0%)	0 (0%)
Clinician (n = 45 )	Sound quality	21 (46.66%)	20 (44.44%)	3 (6.66%)	1 (2.22%)	0 (0%)
	Video quality	21 (46.66%)	22 (48.88%)	1 (2.22%)	1 (2.22%)	0 (0%)

The overall patient experience about the video consultation was positive (Table 4).

**Table 4:** Patient/clinician experience Data are presented as n (%).

		Yes	No	Unsure
Patient (n = 43)	Communicated everything	37 (86.04%)	5 (11.62%)	1 (2.32%)
	Needs met	43 (100%)	0 (0%)	0 (0%)
Clinician (n = 45)	Communicated everything	43 (95.55%)	1 (2.22%)	1 (2.22%)
	Needs met	43 (95.55%)	1 (2.22%)	1 (2.22%)

Overall experience with most of the patients was positive (41/43; 95.34%). Patients were less satisfied than clinicians that they had communicated everything they wanted to (86% versus 95%). All (100%) patients thought that the video telemedicine solution met their needs, but 25% of clinicians thought that the patient experience of a video consultation was worse than a F2F clinic appointment.

Clinician feedback

Overall, 45 times clinicians provided feedback for video consultation. Of the clinicians, 88.88% (40/45) found it easy to join the video call. Majority of them were happy with the sound quality (91.1%; 41/45) and the video quality (95.54%; 43/45) (Table 3); 95.55% (43/45) agreed that they were able to communicate everything and felt that their needs were met (Table 4). Most (42/45; 93.3%) of the clinicians opted to choose video consultation again.

A total of 34 clinicians responded to the second improvement questionnaire. The three major factors identified for introducing video consultations were improved patient experience, successful IT and waiting time (Table 5).

**Table 5:** Clinician responses to the second questionnaire

	Good	Need for Improvement
IT connection and equipment	11	6
Digital records	4	2
Waiting time	9	1
Patient triage	7	6
Location	4	0
Patient experience	21	4

## Discussion

Our study compares clinician and patient experience and satisfaction with video consultation for outpatient during the peak of COVID-19.

During the current COVID-19 pandemic crisis, it was a necessity of the time to bring the change through a model to provide continuity in patient care in such a way to avoid transmission of the deadly virus [2,18]. For this, we utilised Kotter’s model for bringing the change in our Hospital Trust. Kotter’s model was effective in bringing a difference in the institute; none of the research we found to date followed such a model at such speed for the development of a video telemedicine clinic. Our results show that this can be achieved at an accelerated rate, with the most important steps, to recognise the opportunity and assemble the dexterity of a sense of urgency of need without fabricating the reality for future [19].

SWOT analysis results

Strength

Telemedicine has become a crucial and vital policy, with little evidence, especially during times of COVID-19 pandemic type challenging situation. Our results show that telemedicine in hospital outpatient care is dynamic, feasible and convenient to provide enhanced service-based clinical care. One patient stated: “Nothing to suggest - extremely good”. One clinician commented, “Fantastic and so helpful”, whereas others said: “Patient felt safe as no visit to the hospital during COVID-19”. Such a promising benefit of reinforced clinical care has also been observed in recent past [20].

Our clinicians and patients identified outpatient telemedicine as time-saving and stated: “Less time consuming, less likely to have delays”, “Reduce pressure and stress of patients waiting”. This is consistent with patient outcomes reported in primary care [3]. A new previously unacknowledged benefit is the avoidance of viral transmission, particularly relevant internationally during the current COVID-19 pandemic crisis.

Some patients experience an improved multidisciplinary consultation with telemedicine: “Better engagement with MDT members being able to speak with the patient. Top marks!”. Paediatric and young patients found to embrace telemedicine readily because they are using the latest technologies in their routine life. Interestingly, in our collected data, we saw the largest patient group engaged were patients above 51 years of age [15].

Both patients and clinicians found it beneficial in avoiding unnecessary journey as well as further exposure to the viral transmission. One patient stated: “Saved an unnecessary trip to the hospital”. Clinicians also noted such benefit and gave feedback as “No transport to worry about, not having to take off a day from work. There are huge benefits both for the patient but also for the healthcare service in terms of cost…”, “Save patient travel time, especially with the need for transport and mobility issues”. Recently published research also proved that telemedicine saved travel time associated with patient financial benefits [21].

One clinician also highlighted the following distinctive benefit: “Some sharing platform - e.g. to show scans”. “Attend Anywhere” has the option of sharing the screen with the patient where clinicians can share scans or relevant reports with patients [22].

Weaknesses

“It was a big laggy”, said one patient. There is still the possibility of time lag as some may experience difficulties to login, thus technical understanding directly affecting the quality of audio and thus consultation, especially if it is first experience [3,23].

Access to the internet and understanding of technology and system remains factual issues. One clinician also gave feedback about audio quality, “We couldn’t hear dialogue from external”.

Another gave the feedback very clearly at the start of the project as “Patients requiring physical examination are to fall in exclusion criteria for such consultation”. Lack of physical examination is also another major weakness that makes this virtual system not suitable for all patients [6,24]. However, during the era of COVID-19, clinical care has continued with the recognition that physical examination of a patient is gradually being balanced by the reliance on investigations, and therefore this weakness may reduce in importance as time progresses.

Opportunities

Patients and clinicians favoured telemedicine compared to traditional F2F consultation: “I think this is the way forward for follow-up appointments”, “I very much hope that video consultation stay.” Fatehi and Gray found that telemedicine can be used as equivalent to F2F consultation for certain consultation types, with follow up appointments identified as ideal for telemedicine consultation “by default” [25]. Both patients’ and clinicians’ experience shows that it is navigation to a new way of delivering optimal hospital-led specialist care in a way that suits both clinicians and patients.

Our clinicians, while finding opportunities in a video consultation, drew on personal experience and provided recommendations that for telemedicine to be used optimally, all the systems need to be paperless, including prescriptions, requests for investigation and healthcare records. Many healthcare systems are not yet at this level of digital maturity in the NHS and globally; therefore, the opportunities for increasing the use of telemedicine are tremendous. Improving IT functionality and accessibility from the patient side, including a high definition, could further expand the opportunities to deliver remote care, in line with the NHS’s long-term plan [26,27]. Although some inherent weaknesses prevail there, the introduction of telemedicine in COVID-19 has demonstrated that it is relevant for all age groups and all specialities to deliver outpatient care and monitoring.

Threats

We found during this research that there are also some threats to its use. Three clinicians stated: “Quality of video kept breaking off when all members of MDT were on board”, “Seems that more number of people increases the video/audio lag in the system”, “Sometimes no sound, sometimes a huge delay in signal making the consultation and communication very difficult. Patient don’t often have the correct internet speed”.

Unstable connection, low visual and voice quality, and lack of soundproofing can be vital and pose a significant threat to continuing increasing adoption, losing the patient benefit that has been demonstrated. If IT problems prevent a successful video telemedicine appointment, in the UK, reverting to telephone consulting has ensured that no appointments are lost through system or connection problems.

Most patients in this group had their first experience of video consultation; routine use in the near future may help to avoid correctable issues. With time, we believe that there will be a quicker adaptation. Defiance to integrate video telemedicine into the clinical workplace is another threat from clinician’s perspective [20]. We believe that specific consultations may not be appropriate for telemedicine, for example, when there is a need to break bad news, ethical considerations come forward and constitute a more serious threat to its adaptability. IG and software security-related issues may make ethical implementation a crucial and controversial issue. Funding and actual cost gain per patient are the administrative issues that need to be looked at [28].

## Conclusions

Video outpatient consultation (telemedicine) is simple, dynamic, feasible and adaptable. There is a discrepancy between the patients’ positive enthusiasm for telemedicine and clinicians, believing that the patient experience is better with a F2F consultation. This suggests that some of the delays in delivering telemedicine for the UK NHS population arise from clinicians’ incorrect assessment of their patient wishes. This poses the greatest threat to continuing adoption after the crisis phase of COVID-19. Despite some inherent weaknesses and threats to its universal applicability, it has strengths and opportunity of future consultation equivalent to, and at times better than, F2F consultations.

## Appendices

Questionnaire 1: Video Clinic/Telemedicine Questionnaire for Patients

Question 1: What is your age?

o   16 - 30

o   31 - 50

o   51 - 70

o   More than 71

o   Prefer not to say

Question 2: What type of device you used for your video consultation?

o   Mobile

o   Tablet/ iPAD

o   Laptop/computer

Question 3: Which internet browser did you use for your video consultation?

o   Chrome

o   Internet Explorer

o   Edge

o   Safari

o   Other

Question 4: How easy was it for you to join the video call for video consultation?

o   Very easy

o   Easy

o   Neutral

o   Difficult

o   Very difficult

Question 5: How was sound quality during video consultation?

o   Very good

o   Good

o   Average

o   Poor

o   Very poor      

Question 6: How was video quality during video consultation?

o   Very good

o   Good

o   Average

o   Poor

o   Very poor

Question 7: What three things you liked about video consultation?

1.     ___________________________________________

2.     ___________________________________________

3.     ___________________________________________

Question 8: What three things you suggest to improve in video consultation?

1.     ___________________________________________

2.     ___________________________________________

3.     ___________________________________________

Question 9: Do you think that everything was communicated through video consultation?

o   Yes

o   No

o   Unsure

Question 10: Do you think that your needs were met?

o   Yes

o   No

o   Unsure

Question 11: How was your overall experience during video consultation?

o   Very good

o   Good

o   Average

o   Poor

o   Very poor

Question 12: How likely would you choose the video consultation again?

o   Very likely

o   Likely

o   Neither likely nor unlikely

o   Unlikely         

o   Very unlikely

Questionnaire 2: Video Clinic/Telemedicine Questionnaire for Clinicians - Survey 1

Question 1: What is your speciality?

            ____________________

Question 2: How easy was it for you to join the video call for video consultation?

o   Very easy

o   Easy

o   Neutral

o   Difficult

o   Very difficult

Question 3: How was sound quality during video consultation?

o   Very good

o   Good

o   Average

o   Poor

o   Very poor      

Question 4: How was video quality during video consultation?

o   Very good

o   Good

o   Average

o   Poor

o   Very poor

Question 5: What three things you liked about video consultation?

4.     ___________________________________________

5.     ___________________________________________

6.     ___________________________________________

Question 6: What three things you suggest to improve in video consultation?

4.     ___________________________________________

5.     ___________________________________________

6.     ___________________________________________

Question 7: Do you think that everything was communicated through video consultation?

o   Yes

o   No

o   Unsure

Question 8: Do you think that your needs were met?

o   Yes

o   No

o   Unsure

Question 9: How likely would you choose the video consultation again?

o   Very likely

o   Likely

o   Neither likely nor unlikely

o   Unlikely         

o   Very unlikely

Questionnaire 3: Video Clinic/Telemedicine Questionnaire for Clinicians - Survey 2

Question 1: Have you used video consultation at Princess Alexandra Hospital NHS Trust in the last 3 months?

o   Yes

o   No

Question 2: If yes to above, did you find that video consultation changed your experience of a clinic?

o   Yes

o   No

Question 3: What three things would make video consultation better for the doctor or nurse?

1.     ______________________________

2.     ______________________________

3.     ______________________________

Question 4: What three things would make video consultation better for the patient?

1.     ______________________________

2.     ______________________________

3.     ______________________________

Question 5: What do you think about IT connection and equipment available during video consultation?

o   Good

o   Need for improvement

Question 6: What do you think about the availability of digital patient record?

o   Good

o   Need for improvement

Question 7: What do you think about the waiting time for the patient during video consultation?

o   Good

o   Need for improvement

Question 8: What do you think about the triage process for video consultation?

o   Good

o   Need for improvement

Question 9: What do you think about the benefit for a patient with regards to travel during video consultation?

o   Good

o   Need for improvement

Question 10: How do you see patient experience with video consultation?

o   Good

o   Need for improvement
